# Cefadroxil-Induced Radiation Recall Dermatitis: A Case Report and Review of the Literature

**DOI:** 10.7759/cureus.88735

**Published:** 2025-07-25

**Authors:** Kirti Sharma, Bhupesh Parashar, A. Gabriella Wernicke

**Affiliations:** 1 Radiation, Virginia Commonwealth University, Richmond, USA; 2 Radiation Oncology, Northwell Health, New Hyde Park, USA

**Keywords:** antibiotic, cephalosporin-induced, external beam radiation, local reaction, recall reaction

## Abstract

Radiation recall dermatitis (RRD) is a rare phenomenon. There are a few reports in the literature reporting RRD triggered by quinolone administration after external beam radiation therapy (EBRT). We present an unusual case of RRD induced by cefadroxil, which occurred months after completion of EBRT. A patient underwent accelerated partial breast irradiation (APBI) to the right breast, which she finished without any side effects to the skin. She then underwent a left hip replacement and was placed on an antibiotic called cefadroxil 500 mg BID (twice a day), which correlated with a sudden redness in the right breast. The antibiotic was stopped, and her affected breast was treated with clobetasol cream 0.05%, and the patient’s RRD resolved. We review literature emphasizing cefadroxil antibiotic as a causative agent of RRD.

## Introduction

Radiation recall dermatitis (RRD) is an acute inflammatory response that occurs in previously irradiated skin following exposure to certain triggering agents, most commonly chemotherapy agents (e.g., anthracyclines and taxanes) or targeted drug therapies [1,2]. This delayed skin reaction may manifest days, months, or even years after the completion of radiation therapy (RT) and typically presents with erythema, swelling, skin peeling, tenderness, or, in severe cases, blistering and ulceration [3]. While the exact pathophysiology of RRD is not fully understood, potential etiologies include triggering of memory T cells from prior radiation, epithelial stem cell inadequacy or sensitivity, vascular damage, and drug hypersensitivity reactions [4-11]. Management of RRD primarily involves discontinuing the triggering agent, if feasible, symptomatic treatment with topical corticosteroids and emollients, protection from sun exposure, and, in severe cases, the use of oral or intravenous corticosteroids [12].

Despite the widespread use of non-cytotoxic antibiotics for non-malignant conditions in patients with a history of RT, reports of RRD in this context remain scarce in the literature. RRD associated with antibiotic use is rare, and its occurrence in connection with cephalosporins is particularly uncommon. We present a case of a woman who developed RRD following the administration of cefadroxil, a first-generation cephalosporin.

## Case presentation

**Figure 1:**
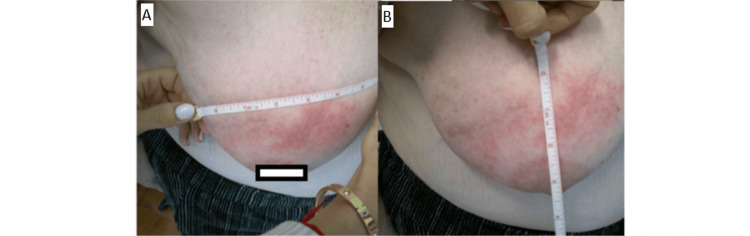
(A, B) Radiation recall reaction (RRD) after cefadroxil 500 mg PO BID for 10 days

**Figure 2:**
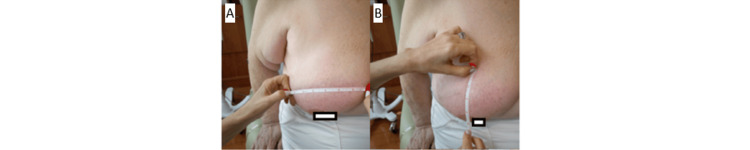
(A, B) One week into treatment of radiation recall dermatitis (RRD) with clobetasol 0.05% cream

**Figure 3:**
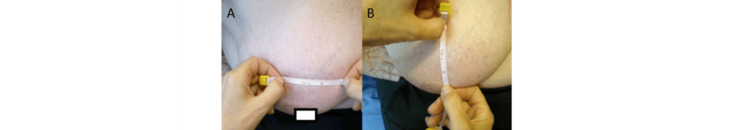
(A, B) Two weeks after clobetasol

This case report is about a 78-year-old patient who underwent right breast partial mastectomy in March 2024, with pathological Stage IC-pT1cN0Mx infiltrating ductal carcinoma of the right breast, with solid papillary features, grade 2, in the upper outer quadrant (11 o'clock) of the right breast, 2 cm from the nipple, ER+/PR+/HER2-. No nodes were submitted or found in the pathology report. The Oncotype score was 4. She completed accelerated partial breast irradiation (APBI) to a total dose of 3,000 cGy in five fractions to the right partial breast in June 2024 with a Radiation Therapy Oncology Group (RTOG) grade 1 acute dermatological reaction, which completely resolved within two weeks after the completion of APBI. In July 2024, the patient was examined by a breast surgeon and had no erythema. On July 31, 2024, the patient underwent a left anterior hip replacement and was placed post-operatively on cefadroxil 500 mg PO BID for 10 days. Within a few days of initiation of the antibiotic, the patient reported a diffuse erythematous skin in the right breast, exactly within the confines of the port of APBI (Figure 1). The medical oncologist planned for tamoxifen 10 mg with a plan to up-titrate to 20 mg as tolerated, but had to hold off on prescribing tamoxifen until after the resolution of the erythema. The patient was not on any additional medications. The patient was seen in the radiation clinic, requested discontinuation of an antibiotic, and was started on clobetasol 0.05% cream BID to be applied to the area of erythema. The patient was followed in the radiation clinic on a weekly basis and was seen to experience gradual resolution of the erythematous reaction (Figures 2, 3). She started tamoxifen and continues to remain reaction-free in her last follow-up, which is nine months after the completion of APBI.

## Discussion

The case above represents an example of RRD, which is defined as an acute skin reaction following drug administration at the location of a previously irradiated area [1,13]. Our patient demonstrates, to our knowledge, the second case of this rare inflammatory skin reaction to the commonly used cephalosporin antibiotic. However, our patient represents the first case to have involved a first-generation cephalosporin: cefadroxil. While the initial account of RRD was published in 1962 upon actinomycin D therapy [14], current literature shows a variety of chemotherapeutic agents as inducing agents, including anthracyclines, taxanes, nucleoside analogues, and antimetabolites-the former two being the most responsible for a majority of RRD cases [2]. Additionally, increasing research supports non-cytotoxic medications to also cause RRD, such as simvastatin [4], tamoxifen [5], cefazolin [6], antitubercular drugs [7], and gatifloxacin [8,9]. There have also been recent reports regarding other non-antibiotic use causing RRD, specifically within the past five years. These case reports demonstrated RRD occurrence from pembrolizumab [15], dabrafenib, trametinib [16], and ribociclib [17] treatment. Our case report focused on antibiotic therapy inducing RRD. Table 1 lists the published reports of antibiotics causing RRD.

**Table 1:** Antibiotic-induced radiation recall case reports RT: radiation therapy

Publication (Ref)	Antibiotic	Site/time after RT	Comments
Kang, 2006 [8]	Gatifloxacin	Pelvic and perineal area 3 years after completion of RT	Resolved 4 days after discontinuation
Jain et al., 2008 [9]	Gatifloxacin	Face and neck 5 days after completion of RT	Topical steroids, pain control, antihistaminics, and cool compresses. Resolved in 8 days
Patel et al., 2020 [18]	Intramuscular ceftriaxone	Neck/6 months after RT completion	Resolved without intervention in 2 days
Garrahy and Forman, 2019 [19]	Nitrofurantoin	Left breast 8 years after RT completion	Resolved after discontinuation within 1 week
Wernicke et al., 2010 [20]	Levofloxacin	Right breast 7 months after completion of RT	Resolved after discontinuation
Vujovic, 2010 [21]	Azithromycin	Left breast 4 years after completion of RT	Resolved in 10 days without any specific intervention

There are many proposed mechanisms for how non-cytotoxic agents can cause RRD, such as epithelial stem cell inadequacy and/or sensitivity, vascular damage, and drug hypersensitivity reactions. Additionally, due to drug specificity, rarity, and speed of onset of RRD, another mechanism hypothesized is idiosyncratic hypersensitivity drug reaction [13]. Due to our patient having a rapid improvement in symptoms upon discontinuation of cefadroxil, the idiosyncratic hypersensitivity drug reaction mechanism is the most likely explanation in this case.

An interplay between RT dose and time before drug exposure can affect both the risk and the rate of onset of RRD [1]. Although no accurate radiation dose threshold has been established, the tumor dose in the reported cases of RRD ranges from 1,000 to 6,120 cGy.

The histology of skin biopsies in patients with RRD includes epidermal dysplasia, keratinocytes with necrosis, increased mitosis, mixed inflammatory infiltrate, and increased p53 staining [11]. Skin reactions generally settle down within a few days of discontinuing the triggering drug. During drug rechallenge, the response can be unpredictable. But dose reduction and steroid use as premedication tend to produce a mild recurrence of RRD [13]. Our patient was counseled to avoid rechallenge with cephalosporins.

Characterizing RRD unequivocally, skin reactions should occur more than seven days after RT completion, and all acute reactions should completely recover before any drug administration [13]. In our patient’s case, the skin reaction was RTOG grade 1 at the conclusion of external beam radiation therapy (EBRT), which negates the contribution of a non-healing acute skin reaction. While photosensitivity is a well-known adverse effect of cephalosporins, we found no published report on the radio-sensitizing potential of first-generation cephalosporins, like cefadroxil.

Cefadroxil is a broad-spectrum cephalosporin antibiotic that has shown a profound impact on the treatment of infectious diseases involving gram-positive and gram-negative organisms [22]. A first-generation cephalosporin, cefadroxil, has bactericidal properties through which it disrupts the enzymatic reactions that are required for bacterial cell wall synthesis. Such a beta-lactam antibiotic works by binding and deactivating penicillin-binding proteins (PBPs), which are enzymes at the last stage of bacterial cell wall synthesis [23]. The pharmacokinetic profile of cefadroxil includes its long serum half-life and slow rate of excretion, which allows patients to be on a once- or twice-daily dosage regimen. In addition, cefadroxil is absorbed within the gastrointestinal tract upon oral administration. While the presence of food may lower the rate of diffusion of some antibiotics across the mucosal barrier, studies have shown that taking cefadroxil with food does not affect its absorption [24]. Due to its water-soluble and some lipid-soluble characteristics, cefadroxil is widely distributed in body tissues and fluids, including tonsils, lungs, liver, gallbladder, bone, muscle, prostate, and gynecological tissues. Body fluid distribution includes pleural fluid, bile, sputum, breast milk, skin blisters, and amniotic fluid. In a patient with normal renal function, cefadroxil does not metabolize and would be cleared primarily through renal processes [23]. As mentioned earlier, cefadroxil has been indicated for the treatment of infectious diseases, including urinary tract infections, pharyngitis, tonsillitis, skin and soft tissue infections, and osteomyelitis [23,25]. But there are also side effects associated with cefadroxil treatment, mostly gastrointestinal disturbances and hypersensitivity reactions. These adverse reactions include nausea, vomiting, diarrhea, allergic rash, urticaria, and dermatitis [23].

## Conclusions

While the occurrence of RRD due to cephalosporin is quite rare, clinicians may encounter more cases because of the increasing use of this drug. Cefadroxil may cause an idiosyncratic hypersensitivity reaction, leading to RRD. The skin reactions seen after such common antibiotics can have a negative impact on the quality of life for patients after breast-conserving treatment. Cefradoxil-induced RRD was mild and resolved on discontinuation of antibiotics plus local symptomatic treatment. Given the rarity of RRD with cephalosporins, it is difficult to prevent its occurrence, but early recognition and proper management are important for a favorable outcome, and RRD should be considered a differential diagnosis when an unusually significant and localized skin reaction occurs at a site of previous RT and after cefadroxil administration. There needs to be additional research on the mechanism of RRD secondary to chemotherapeutic agents, antibiotics, and additional pharmacological agents.
